# Activation of cellular immunity and marked inhibition of liver cancer in a mouse model following gene therapy and tumor expression of GM-SCF, IL-21, and Rae-1

**DOI:** 10.1186/1476-4598-12-166

**Published:** 2013-12-18

**Authors:** Mingrong Cheng, Kangkang Zhi, Xiaoyan Gao, Bing He, Yingchun Li, Jiang Han, Zhiping Zhang, Yan Wu

**Affiliations:** 1 Department of General Surgery, Pudong New Area District Zhoupu Hospital, Shanghai 201318, China; 2 Department of Endoscopy, Pudong New Area District Zhoupu Hospital, Shanghai 201318, China; 3 Department of Vascular & Endovascular Surgery, Changzheng Hospital, the Second Military Medical University, Shanghai 200003, China; 4 Department of Plastic Surgery, Pudong New Area District Zhoupu Hospital, Shanghai 201318, China; 5 Department of General Surgery, Shanghai Fifth People’s Hospital, Fudan University, Shanghai 200240, China

**Keywords:** Gene therapy, Natural killer cells, Cytotoxic T lymphocytes, Liver cancer, Immune escape, Cell-mediated immunity

## Abstract

**Background:**

Cancer is both a systemic and a genetic disease. The pathogenesis of cancer might be related to dampened immunity. Host immunity recognizes nascent malignant cells – a process referred to as immune surveillance. Augmenting immune surveillance and suppressing immune escape are crucial in tumor immunotherapy.

**Methods:**

A recombinant plasmid capable of co-expressing granulocyte-macrophage colony- stimulating factor (GM-SCF), interleukin-21 (IL-21), and retinoic acid early transcription factor-1 (Rae-1) was constructed, and its effects determined in a mouse model of subcutaneous liver cancer. Serum specimens were assayed for IL-2 and INF-γ by ELISA. Liver cancer specimens were isolated for Rae-1 expression by RT-PCR and Western blot, and splenocytes were analyzed by flow cytometry.

**Results:**

The recombinant plasmid inhibited the growth of liver cancer and prolonged survival of tumor-loaded mice. Activation of host immunity might have contributed to this effect by promoting increased numbers and cytotoxicity of natural killer (NK) cells and cytotoxic T lymphocytes (CTL) following expression of GM-SCF, IL-21, and Rae-1. By contrast, the frequency of regulatory T cells was decreased, Consequently, activated CTL and NK cells enhanced their secretion of INF-γ, which promoted cytotoxicity of NK cells and CTL. Moreover, active CTL showed dramatic secretion of IL-2, which stimulates CTL. The recombinant expression plasmid also augmented Rae-1 expression by liver cancer cells. Rae-1 receptor expressing CTL and NK cells removed liver cancer.

**Conclusions:**

The recombinant expression plasmid inhibited liver cancer by a mechanism that involved activation of cell-mediated immunity and Rae-1 in liver cancer.

## Introduction

In the 1950s, Burnet proposed that the immune system could recognize nascent malignant transformed cells in a process known today as immune surveillance [1]. Anti-tumor immunity to aberrant self-antigen (tumor antigen) is prioritized in cellular immunity, and requires the interplay of a variety of accessory and major effector cells including CD8+ and CD4+ T cells [2,3]. When tumor cells fail to express an antigenic epitope, defects might occur in antigen processing, modulation, or tumor antigen loss. There might also be a lack of major histocompatibility complex class I (MHC- I) expression, functional co-stimulatory molecule expression, inhibition of tumor cell expression, and tumor cell expression of apoptotic regulatory ligands. In addition, there could be general immunodeficiency and immunosuppression of natural killer (NK) cell and cytotoxic T cell (CTL) activity. Collectively. each of these pathways could promote immune escape of the tumor target [4-6]. Therefore, cancer cells attempt to use multiple mechanisms to escape recognition by and attack by host immunity to promote their survival and proliferative capability. However, immune escape can be avoided in one of two ways. Firstly host immunity could be stimulated, especially in the context of anti-tumor CTL and NK cells. Secondly the expression of antibodies or ligands that are recognized by host immunity in cancer cells might be augmented, and examples of this include natural killer group 2D (NKG2D).

NKG2D is a C-type lectin-like activating receptor that is present on the surface of NK cells with a stress-inducible ligand of MHC- I class molecules A/B (MIC A/B) [7]. The retinoic acid early transcription factor-1 (Rae-1) is one of the most frequently studied mouse NKG2D ligands. Rae-1 is detected in many mouse cancer cells. Similar to MICA and MICB ligands of NKG2D in the human immune system, Rae-1 is highly expressed under stressful environments (e.g., in response to heat shock, ultraviolet light, virus infection, malignant transformation, and exposure to carcinogenic substances). The affinity of Rae-1 for NKG2D is tens or hundreds of times greater than that of the killer inhibitory receptor (KIR) [8]. Rae-1 is not expressed in normal cells but is restricted to expression by epithelial cancer cells. Expression of Rae-1 is combined with NKG2D and mediated by the DAP10 pathway (the “self-induction hypothesis”), making cancer cell expression of Rae-1 easily identified by immune cells and hence removal of cancer cells that express it [9]. Thus NK and CD8 + T cells are capable of removing cancer cells that highly express NKG2D ligands [10]. *In vitro* experiments showed that induced expression of NKG2D ligands following transfection of cancer cells and antibody blocking significantly enhance tumor cell susceptibility to NK cells. Perhaps of greater relevance is the observation that subcutaneous injection of cancer cells containing the transfected NKG2D gene in mice induces potent tumoricidal immune responses and significant dampening of tumor cell growth [10]. Consequently, immune cells easily identify tumor cells that highly express Rae-1.

Others have shown that gene expression of both GM-CSF and IL-21 can significantly inhibit tumors and activate host immunity including CTL and NK cell activation [11,12]. Previously, we studied recombinant plasmids that expressed both GM-CSF and IL-21 in a mouse model of orthotopic liver cancer by intravenous tail vein injection [13]. This construct markedly blocked the growth of tumors and enhanced both NK cell and CTL activity.

The current study focuses on stimulating either cell-mediated immune activation including CTL and NK cells [14], or enhancing the expression of molecules like Rae-1 that are expressed by tumor cells and subsequently identified by host immunity [15]. Few reports have shown the effects of attempting to simultaneously increase immune activation and the molecules identified by immune surveying cells. In this study, we propose an immune escape inhibitory system that is based on the immune escape hypothesis and our previously published work.

## Materials and methods

### Reagents and instruments

Methyl Thiazolyl Tetrazolium (MTT) was obtained from Sigma Ltd, Shanghai, China. Plasmid maxi preparation kits were obtained from Promega (Beijing) Biotech Co., Ltd., Beijing, China. Interferon (IFN-γ), interleukin-2 (IL-2), and enzyme-linked immunosorbent assay (ELISA) kits were obtained from Santa Cruz Biotechnology, Santa Cruz, CA, USA. Fluorescent-labeled antibodies of fluorescein isothiocyanate (FITC)-anti-mouse CD3, PE-anti-mouse CD4, PE-anti mouse CD8, FITC-anti-mouse CD25, Alexa 647-anti mouse Foxp3, FITC-anti-mouse CD11b, and PE-anti-mouse CD27 were provided by BD Bioscience, San Jose, CA, USA.

### Mice and cell-lines

Balb/c mice (male, 7 wk old, weighing 20 g, and specific pathogen free (SPF)) were obtained from the Animal Center of Fudang University (Shanghai, China). Hepatic cancer cells (H22) were provided by the China Center for Type Culture Collection (CCTCC, Wuhan, China). The target cell-line YAC-1 of natural killer (NK) cell origin was routinely cultured in the immunology laboratory of Shanghai Fudan University. The selected culture medium was RPMI 1640 and was obtained from the Sigma Chemical Company. The ethics committee of Shanghai Zhoupu Hospital (Shanghai, China) and Fudan University approved the mouse model experiments described in this report.

### Construction of the recombinant plasmid of pGM-CSF-GFP -IRES-IL-21-Rae-1

The genes for both GM-CSF and IL-21 were obtained from the spleens of mice. Rae-1 and GFP were chemically synthesized. The polymerase chain reaction (PCR) primers were designed and synthesized according to the genetic coding sequence (CDS) for both GM-CSF and IL-21 (Table 1). The enzyme cleavage sites targeted by Xhol and EcoRI were added to the 5′ and 3′ ends of the GM-CSF gene, respectively. EcoRI and MluI cleavage sites were added to the 5′ and 3′ ends of the GFP gene, respectively. Xba1 and Sal1 cleavage sites were added to the 5′ and 3′ end of the Rae-1 gene, respectively. Sal1 and Not1 cleavage sites were added to the 5′ and 3′ ends of the IL-21 gene, respectively. GFP segmentation was connected with a destination vector pIRES, forming a plasmid pGFP-IRES, which was added to the GM-CSF gene to form a vector referred to as pGM-CSF-GFP-IRES, followed by insertion of the PCR products of the Rae-1 gene to obtain a new vector referred to as pGM-CSF-GFP-IRES-Rae-1. The IL-21 digested segments were inserted to form the pGM-CSF-GFP-IRES-Rae-1 expression construct, to form a eukaryotic expression vector referred to as pGM-CSF-GFP-IRES-Rae-1-IL-21. In the process, GM-CSF-GFP was used for MCS A and Rae-1-IL-21 was used for MCS B.

**Table 1:** Showing the primer sequences used to amplify the genes for GM-CSF, GFP, Rae-1, and IL-21

**Genes**	**Primers**
GM-CSF-XhoI-F	CAGAT*CTCGAG*GCCACCATGTGGCTGCAGAATTTACTTTTC
GM-CSF-EcoRI-R	GTCA*GAATTC*CCATTTTTGGCCTGGTTTTTTG
GFP-EcoRI-F	GTCA*GAATTC*ATGGTGAGCAAGGGCGAG
GFP-MluI-R	ACTTA*ACGCGT*TTACTTGTACAGCTCGTCCATGC
Rae-1-Xba1-F	GTACAT*TCTAGA*GCCACCATGAGTCTGTTTGGATCAACCTCT
Rae-1-Sal1-R	GAAT*GTCGAC*GTATTTCTTATTCCTTGGCTTTAGCT
IL-21-Sal1-F	GTCA*GTCGAC*GCCACCATGGAGAGGACCCTTGTCTGTC
IL-21-Not1-R	AATT*GCGGCCGC*CTAGGAGAGATGCTGATGAATCATC

Note: Italics indicates the enzyme cleavage sites for XhoI and EcoRI, EcoRI and MluI, Xba1and Sal1, and Sal1and Not1.

### A mouse model of subcutaneous liver cancer

The hepatic cancer cell-line H22 was adoptively transferred into mice to construct a mouse model of subcutaneous liver cancer. The mice were sacrificed to harvest their tumor tissues. Fresh tumors with vigorous growth were selected and prepared as a tumor single-cell suspension (at a density of 6 × 10^7^ cells mL^-1^). The suspension (50 μL) was injected into the armpit of the right forelimb of experimental mice using a 1 mL syringe.

### Recombinant plasmid treatment in a mouse model of subcutaneous liver cancer

On the 5th day after the mouse model was established, mice were randomly divided into six groups of 10 mice per group including control, IRES/GFP, IRES/IL-21, IRES/GM-SCF, IRES/GM-SCF-IL-21 and IRES/combination. In the control group, mice were injected with 200 μL phosphate buffered saline (PBS). Additionally, 200 μL PBS containing 100 μg of the recombinant plasmid was injected to the mouse tumors of each group (IRES/GF, IRES/IL-21, IRES/GM-SCF, IRES/GM-SCF-IL-21 and IRES/combination). The injection was repeated once a day for five continuous days. After ten days, the mice in each group were sacrificed by overdosing of the anesthesic agents. The liver cancer tissues of each mouse in each group were separated from the mouse by a conventional technique and made into specimens suitable for determination of their weights and volumes. Tumor volumes were calculated by the formula: Tumor volume (cm^3^) = 0.523 × L × W^2^. Here, L (cm) and W (cm) indicated the length and width of the tumors respectively as measured by a Vernier caliper. Gene and protein expression of GFP, Rae-1, GM-SCF and IL-21 were then detected in the tumor tissues by RT-PCR and Western blot analysis of each group. The remaining experimental models of 13 mice per group were used for survival analysis.

### ELISA detection of IL-2 and INF-γ

Mouse serum specimens were isolated in each group and kept at -20°C for determination of IL-2 and INF-γ content. For ELISA detection, serum specimens were thawed at 37°C in an incubator, followed by dilution with double distilled water to 500 μL. Meanwhile, the IL-2 and INF-γ standards were diluted to 8000 μg L^-1^. In a 96-well microtiter plate, each well then received 150 μL diluted specimens, and then 50 μL standards or specimens were added for 15 min. They were oscillated evenly and incubated for 2 h at room temperature. The liquid in the wells was removed and the wells washed with 400 μL of detergent-containing buffer four times, followed by the addition of horseradish peroxidase-conjugated IL-2 and INF-γ (200 μL), then oscillated evenly, and incubated for 2 h at room temperature. Next, the liquid was removed and washed with 400 μL detergent-containing bugger four times. An equal amount of developer A and B were evenly mixed, which were added with 200 μL of the enzyme labeled antibody, and then incubated at room temperature in the dark for 30 min. Next, 50 μL of stop buffer was added to complete the enzyme reaction. The specimens were immediately detected using spectrophotometry by measuring its optical density at a wavelength of 450 nm. Samples and standards were run in triplicate, and the sensitivity of the assays was determined to be 0.1 units/mL for both IL-2 and INF-γ.

### Reverse transcription-polymerase chain reaction (RT-PCR)

Total cellular RNA was determined by the Trizol method. The reverse transcription was conducted using 1 μL RNA and 0.5 μL AMV reverse transcriptase, 2.5 μL cDNA as a template for PCR amplification, 0.1 μL polymerase Ex Taq HS, 0.1 μL sense primer and 0.1 μL antisense primer. The reaction conditions were: 94°C initial denaturation for 2 min, 94°C denaturation for 40 s, 50-65°C annealing for 40 s, 72°C extension for 1 min, and a total of 35 cycles, and 72°C extension for 5 min for one cycle. The PCR products were kept frozen at -20°C. The glyceraldehyde-phosphate dehydrogenase (GAPDH) gene was used as an internal housekeeping control and amplified as described above. The shRNA target fragments and 6 μL of the PCR products of the internal control GAPDH were separated on a 2% agarose gel and by electrophoresis at 120 V and 100 mA for 30 min. After electrophoresis, the gel was placed in ethidium bromide (EB) staining solution for 5 min. The positive PCR reactions showed clear amplification segments as expected. Quantity-One software (Bio-Rad Laboratories Inc, Hercules, CA, USA) was used for analysis of electrophoresis data, and gray-scale scanning. By comparison against the gray values of GAPDH, we obtained relative values of the mRNA products in the sample target genes.

### Western blot analysis of relative protein expression

The extraction was performed in order to harvest sufficient proteins for 12% w/v sodium lauryl sulfate polyacrylamide gel electrophoresis (SDS-PAGE). Before placing samples on the separating gel, proteins underwent electrophoresis at 80 V and then at 120 V until its front was about 1 cm from the bottom of the gel. Polyvinylidene fluoride fibre (PVDF) membranes were immersed in methanol for 5 min, then transferred to a buffer solution (pH 8.3, 25 mmol L^-1^ Tris–HCl, 192 mmol L^-1^ glycine, and 20% methanol) for 10 min at room temperature. Proteins were transferred onto PVDF membranes at 100 V for 70 min, and then blocked with 5% bovine serum albumin/PBS by incubating the membrane overnight at 4°C, and then reacting the membrane with diluted primary antibodies (1:2000) for 3 min at room temperature, rinsing in 0.05% PBS-Tween 20 three times for 10 min each. Membranes were then reacted with diluted secondary antibodies (1:8000) for 3 h targeted against the membrane-bound protein-primary antibody complexes with rinsing in 0.05% PBS-Tween 20 three times for 10 min each, followed by detection in ECL reagent. Equal parts of solutions A and B in the ECL kit were mixed, and reacted with the membrane for 1 min. Proteins were developed and photographed by a gel imaging system. The film scanning of the protein was performed using an Image J version 1.44 software program to obtain average density values (ADV). The relative absorbance value was calculated as a ratio of ADV and GAPDA.

### Flow cytometry

Mice spleens were harvested, carefully ground, and filtered against mesh screens to obtain single-cell suspensions, which were incubated in RPMI 1640. Cells were seeded into a 96-well microtiter plate, followed by centrifugation at 300 *g* for 5 min. Then, cell pellets were retained and washed 2–3 times with 200 μL of PBS. The washed cells were mixed with a blocking agent and incubated under gentle shaking at 4°C for 60 min, followed by washing 2–3 times with 200 μL of PBS. Diluted antibodies (anti-CD3, anti-CD4, anti-CD8, anti-CD25, anti-CD19, anti-CD11b, anti-CD27 and anti-Foxp3 antibodies) were added to their respective wells, incubated at 4°C under gentle shaking for 60 min, and washed 2–3 times with 200 μL of PBS. Then, cells were resuspended in 200 μL of cold PBS and kept in the dark for determination of the total numbers of CD3+ T cells, Th cells of the CD3^+^ CD4^+^ phenotype, CTL cells of the CD3^+^ CD8^+^ phenotype, B cells of the CD3^-^ CD19^+^ phenotype, NK cells of the CD11b^+^ CD27^+^ phenotype, and Treg cells expressing CD4^+^ CD25^+^ Foxp3^+^ by flow cytometry (BD Biosciences, Franklin Lake, NJ, USA).

### MTT detection of CTL and NK activity

Using the random number method, three mice models were detected by MTT in each group. Spleen cells (NK and CTL cells) were enumerated by flow cytometry. Their densities were then adjusted to 1×10^6^/mL. YAC-1 and H22 were used as target cells for NK and CTL reactivity, respectively. Cells were incubated in RPMI 1640 containing 10% fetal bovine serum at 37°C in an atmosphere of 5% CO_2_ in air for 48 h. Cell density was adjusted to 1 × 10^5^/mL. Three mice were randomly selected in each group for detection of CTL and NK cell activity. One hundred μL of spleen and target cells (at an effector:target cell ratio of 10:1) were added to a 96-well staining plate. After 18 h of incubation, MTT (20 μL) was added to each well and incubated for an additional 4 h. After centrifugation, the supernatant was removed, and dimethyl sulfoxide (DMSO) (200 μL) was added and agitated for 20 min. Absorbance was determined at a wavelength of 570 nm (A_570_). The activity of CTL and NK cells were calculated as follows:

$$\begin{array}{ll} \text{Activity}= & \left(1-\left(A_{570}\mathrm{of}\;\text{effector}-\text{target}\;\text{cells}-A_{570}\mathrm{of}\;\text{effector}\;\text{cells}\right)\right)\\ & \left(/A_{570}\mathrm{of}\;\text{target}\;\text{cells}\right)\times 100\% \end{array}$$

### Observation of NK cells and CTL by fluorescence microscopy

NK cells and CTL were detected by flow cytometry according to the method described above. NK cells or CTL were permitted to attach to the coverslips in 6-well plates, following which, the medium was removed, the plates were rinsed twice with PBS, and 2 mL 4% paraformaldehyde was added to each well. Plates were incubated at room temperature for 20 min, rinsed with 2 mL PBS three times for 5 min every time. Cells were permeabilized with 2 mL 0.1% Triton X-100 in phosphate buffered saline solution at 4°C for 10 min, aspirated, and cells were rinsed in PBS for 5 min at room temperature. Next, non-specific interactions were blocked with 4% bovine serum albumin in phosphate buffered saline solution at 37°C for 30 min, and primary antibody solution was added and incubated at 4°C overnight. The primary antibody solution was removed, washed in PBS for 5 min, add secondary antibody solution was added to the cells and incubated at room temperature for 1 hr. Cells were washed in PBS three times for 5 min each, following which anti-fade DAPI solution was added if needed. Finally NK cells or CTL were observed by fluorescence microscopy.

### Data analysis

All data were expressed as mean ± standard deviation (SD). One-way analysis of variance (ANOVA) and the least significance difference (LSD) test were used for inter-group comparisons of the data. Kaplan-Meier survival plots were used for calculation of survival data. For all analyses, a probability less than an alpha value of 0.05 (*P* < 0.05) were considered significantly different among groups.

## Results

### Identification of the new vector pGM-CSF-GFP-IRES-Rae-1-IL-21

**Figure 1:**
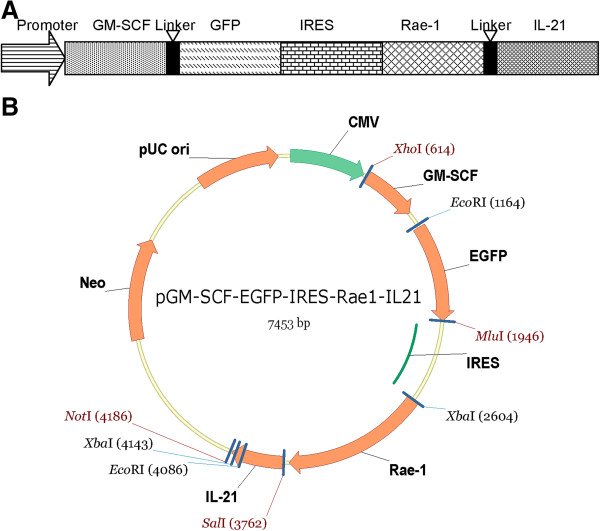
**Design and construction of the new expression vector pGM-CSF-GFP- IRES-Rae-1-IL-21. A**: Design and construction of the immune escape inhibitory system; **B**: the map of the pIRES expression vector.

According to the pIRES vector map (Figure 1A-B), the genes of GFP, IL-21, GM-CSF and Rae-1 were built into the empty plasmid and the new vector pGM-CSF-GFP-IRES-Rae-1-IL-21 was constructed. The positive clones were selected by restriction endonuclease, and the precise clones were confirmed by DNA sequence analyses and end-use sequences. The band of the GFP product (720 bp) was obtained by cutting pGM-CSF-GFP-IRES-Rae-1-IL-21 by EcoRI and MluI, and had the same size as the target gene. Likewise, the band of the GM-CSF product (425 bp) was obtained by cutting pGM-CSF-GFP-IRES-Rae-1-IL-21 by XhoI and EcoRI, and had the same size as the target gene. The band of Rae-1 (1106 bp) that was obtained by cutting pGM-CSF-GFP-IRES-Rae-1-IL-21 with Xba1 and Sal1 had the same size as the target gene. The band of the IL-21 (441 bp) product was obtained by cutting pGM-CSF- GFP-IRES-Rae-1-IL-21 by Sal1and Not1, and had the same size as the IL-21 target gene. The obtained genes of GFP, GM-CSF, Rae-1, and IL-21 were identical with the original sequences as defined in Genbank, and confirmed that the new vector was successfully constructed (DNA sequence analysis data not shown).

### Inhibitory effect of pGM-CSF-GFP-IRES-Rae-1-IL-21 on mouse liver cancer

**Figure 2:**
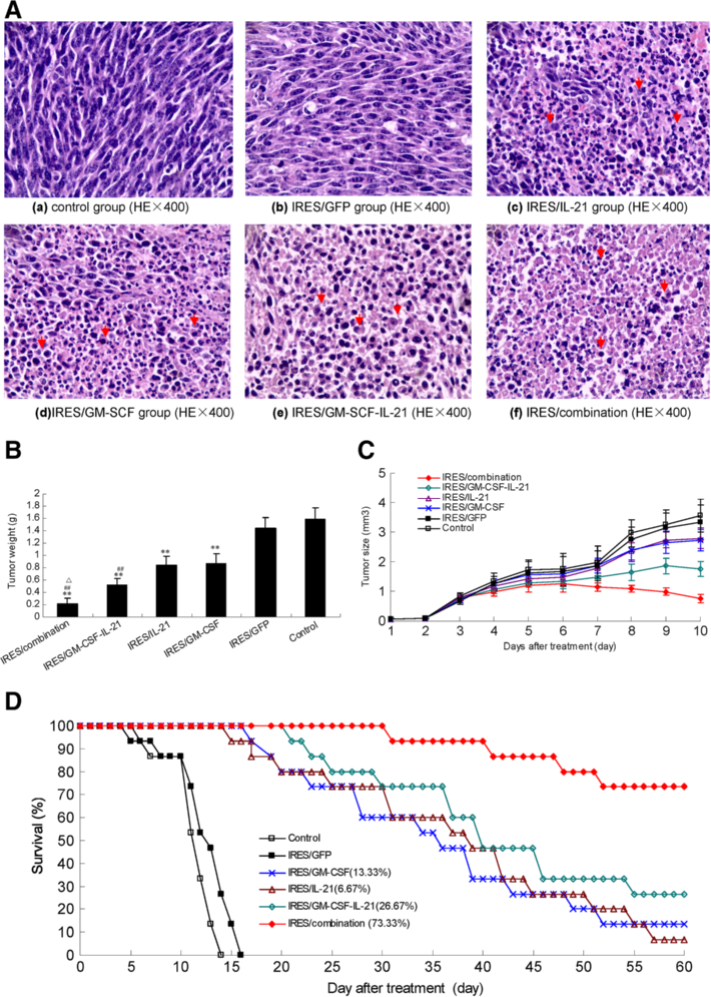
**Inhibitory effects of different expression plasmids on liver cancer in a mouse model.** The differential plasmid treatments were: Control, pGFP-IRES, pGM-CSF-IRES, pIL-21-IRES, pGM-CSF-IRES-IL-21, and pGM-CSF-GFP-IRES-Rae-1-IL-21. Tumor-bearing mice were sacrificed 10 d after treatment and histological sections were made from various tumor tissues. **A**; Histopathology of tumor tissues in mouse liver cancer (HE × 400 magnification). (a) and (b) shows histopathological tumor tissue sections in mice treated with PBS or pGFP-IRES, wherein the tumor cells grew actively. (c) and (d) shows histopathological sections from the tumor tissue of tumor-bearing mice treated with pGM-CSF-IRES or pIL-21-IRES, wherein the tumor cells grew slowly, and only few immunocytes were seen infiltrating the tumor tissue. (e) showing histopathological sections from the tumor tissues of tumor-bearing mice treated with pGM-CSF-IRES-IL-21. Infiltration of immunocytes increased appreciably and necrotic tumor cells were noticeable in the tumor tissue. (f) showing histopathological sections of the tumor tissues in tumor-bearing mice treated with pGM-CSF-GFP-IRES-Rae-1-IL-21. Here, both monocytes and neutrophils infiltrated the tumor, and there was evidence of mass necrosis seen in the tumor tissue. Note that the red arrow indicates inflammatory cells. **B**; showing the tumor weights (expressed as mean ± standard deviation) of mice in each group 10 d after treatment (n = 10). **C**; tumor volumes of mice (expressed as mean ± standard deviation) in each group 1–10 d after treatment (n = 10). **D**; showing the survival time of the mice in each group (n = 13) that were analyzed by the Kaplan-Meier approach. Note: ^**^P < 0.01, as compared with the control. ^##^P < 0.01, as compared with the IRES/GM-SCF or IRES/IL21 expression constructs. ^△^P < 0.05, as compared with the IRES/GM-SCF-IL-21 expression construct.

After 10 days of treatment, mice in each group were sacrificed by overdosing of anesthesia and mice were then routinely dissected for their livers cancer specimens. The histological analyses were performed on the tumor sites, and revealed that the IRES/combination group were infiltrated by massive numbers of monocytes and neutrophils, the tumors had obviously shrunk, experienced ordered rarefaction, and had degenerated (Figure 2A(f)). In the IRES/GM- SCF-IL-21 group, infiltration of immunocytes had increased appreciably and necrotic tumor cells were noticeable in the tumor tissue (Figure 2A(e)). In the IRES/GM-SCF or IRES/IL-21 group the tumor cells grew slowly, and there were few immunocytes infiltrating the tumor tissues (Figure 2A(c-d)). By contrast, the active growth of the tumor cells, and obvious nuclear division or diverse nucleic type, was found in both the control and the IRES/GFP groups (Figure 2A(a-b)).

Histological analyses suggested that the GM-SCF, IL-21, and Rae-1 genes alone or in combination induced a cellular immune response against H22 tumor cells. The specimens were weighed and the results described (Figure 2B). The tumor weight averaged 0.207, 0.522, 0.873, 0.843, 1.439, and 1.591 g for IRES/combination, IRES/GM-SCF-IL-21, IRES/GM-SCF, IRES/IL-21, IRES/GFP, and the control group, respectively, which were significantly different (P < 0.01). The tumor was also significantly (P < 0.01) lighter in the IRES/GM-SCF-IL-21 group than was found in either the IRES/GM-SCF or IRES/IL-21 groups and was not significantly different (P > 0.05) between either the IRES/GM-SCF group or the IRES/IL-21 group.

The tumor was significantly heavier in the control group and lightest in the IRES/combination among the six groups (P < 0.05). By 1–5 days after treatment, the tumor volume had gradually increased in all groups (Figure 2C). After 6 days, the tumor volume had gradually decreased in the IRES/combination group, and was marginally suppressed in the IRES/GM-SCF-IL-21, IRES/GM-SCF, and IRES/IL-21 groups, but had increased rapidly in both the IRES/GFP and control groups. At the conclusion of treatments, tumors were significantly smaller (P < 0.01) in the IRES/GM-SCF-IL-21 group than was found in either the IRES/GM-SCF or IRES/IL-21 groups but this was not significantly different (P > 0.05). The tumor volume of the mice was highest in both the IRES/GFP group and the control group (P < 0.01) and smallest in the IRES/combination group (P < 0.01).

The mice models of subcutaneous liver cancer were randomly divided into six groups with 13 mice in each group and were treated with the procedure described above to record their survival rates by the Kaplan-Meier survival method. Mouse survival was found to decline, with the first deaths seen at 6, 5, 17, 15, and 21 days after treatment in the control, IRES/GFP, IRES/GM-SCF, IRES/IL-21, and IRES/GM-SCF-IL-21 groups respectively (Figure 2D). All mice had demised by 14 and 16 days after treatment in the control and IRES/GFP groups, respectively. There were 2, 1, and 11 mice surviving 60 days after treatment in the IRES/GM-SCF, IRES/IL-21, and IRES/GM-SCF-IL-21 groups respectively. The survival rates of mice were 73.33%, 13.33%, and 6.67% at 60 days of treatment for groups IRES/GM-SCF-IL-21, IRES/GM-SCF and IRES/IL-21, respectively. The survival rate of the mice was significantly higher in the IRES/GM-SCF-IL-21 than was found in the other groups.

### Effect of pGM-CSF-GFP-IRES-Rae-1-IL-21 on IL-2 and INF-γ levels

**Figure 3:**
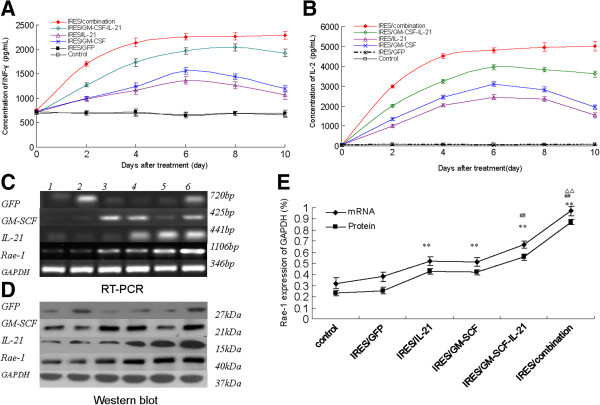
**IL-2 and INF-γ levels in mouse serum and expression of GPF, GM-SCF, IL-21 and Rae-1 in liver cancer tissue in each treatment group.** Effect of the plasmids on IL-2 and INF-γ levels in mouse serum of each group that included: control, pGFP-IRES, pGM-CSF-IRES, pIL21-IRES, pGM-CSF-IRES-IL21, and pGM-CSF-GFP-IRES-Rae-1-IL-21. At 10d after treatment, mouse serum was quantified by ELISA for IL-2 levels and INF-γ levels. Tumor tissue was assessed by RT-PCR and Western blot to determine GPF, GM-SCF, IL-21 and Rae-1 expression. **A**: IL-2 levels were detected and expressed as mean ± standard deviation (n = 3); **B**: INF-γ levels were tested by ELISA and expressed as mean ± standard deviation (n = 3); **C**: RT-PCR analysis of GPF, GM-SCF, IL-21 and Rae-1 in liver cancer tissue (n = 3). **D**: Western blot analysis of GPF, GM-SCF, IL-21 and Rae-1 in liver cancer tissue (n = 3). **D**: Western blot analysis of GPF, GM-SCF, IL-21 and Rae-1 in liver cancer tissue (n = 3). **E**: relative values of Rae-1 protein or mRNA expression in liver cancer tissue of each group, expressed as mean ± standard deviation (n = 3). Note: Lane 1, control. Lane 2, pIRES/GFP. Lane 3, pIRES/IL2. Lane 4, pIRES/GM-SCF. Lane 5, pIRES/GM-SCF-IL21. Lane 6, pIRES/combination. ^*^ P < 0.05 and ^**^ P < 0.01, as compared with control. ^#^ P < 0.05 and ^##^ P < 0.01, as compared with IRES/GM-SCF and IRES/IL21. ^△^ P < 0.05 and ^△△^ P < 0.01, as compared with IRES/GM-SCF-IL21.

The levels of IL-2 and INF-γ of mice 1–6 days after treatment gradually increased in the IRES/combination groups, including IRES/GM-SCF-IL-21, IRES/GM-SCF and IRES/IL-21 (Figure 3A-B). They were highest in the IRES/combination group and lowest (P < 0.01) in the IRES/GM-SCF and IRES/IL-21 groups, with the IRES/GM-SCF-IL-21 group showing intermediate levels. By 6–10 days after treatment, IL-2 and INF-γ levels had stably increased in the IRES/combination groups, but had gradually decreased in the IRES/GM-SCF-IL-21, IRES/GM-SCF and IRES/IL-21 groups. At the end of treatment, IL-2 and INF-γ levels were significantly higher in the IRES/GM-SCF-IL-21 group than were found in either the IRES/GM-SCF group or IRES/IL-21 group (P < 0.01), which were also significantly higher than either the IRES/GFP or control groups (P < 0.01). The levels of IL-2 and INF-γ were highest in the IRES/combination group (P < 0.01) and not significantly (P > 0.05) different among the IRES/GM-SCF, IRES/IL-21, IRES/GFP, and control groups. By 1–10 days after treatment, IL-2 and INF-γ levels remained unaltered in the IRES/GFP and control groups.

### Effect of pGM-CSF-GFP-IRES-Rae-1-IL-21 on expression of Rae-1 and protein in liver cancer tissue

RT-PCR and Western blot assays were used to detect liver cancer tissues and to analyze the effect of GFP, GM-SCF, IL-21 and Rae-1 constructs and protein expression in liver cancer tissue. The genes and protein expressions of GM-SCF in IRES/GM-SCF, IRES/GM-SCF- IL21 and the IRES/combination group were significantly enhanced (Figure 3C-D). Similarly, the expression of IL-21 in IRES/IL21, IRES/GM-SCF-IL21 and IRES/combination groups were significantly increased (Figure 3C-D). Moreover, Rae-1 mRNA and protein expression in the IRES/combination group was the highest, and the gene and protein expression of GFP were markedly higher in the IRES/GFP and IRES/combination group. These observations informed us that the recombinant plasmids were successful constructed and transfected *in vivo* and were stably expressed. Expression of Rae-1 and protein were significantly (P < 0.01) different among the six groups (Figure 3C-E). Rae-1 was also significantly (P < 0.01) higher in the IRES/combination group as compared with the IRES/GM-SCF-IL-21, IRES/IL-21, or IRES/GM-SCF groups, and was significantly (P < 0.01) higher in the IRES/GM-SCF-IL-21 group as compared with the IRES/IL-21 or IRES/GM-SCF groups. Rae-1 expression was also significantly (P < 0.01) higher in the IRES/IL-21 or IRES/GM-SCF groups than in the IRES/GFP and control groups. However, expression of Rae-1 was not significantly (P > 0.05) different between the IRES/IL-21 and IRES/GM-SCF groups, or when compared with either the IRES/GFP and control groups.

### Effect of pGM-CSF-GFP-IRES-Rae-1-IL-21 on the frequencies of NK and CTL cells, cellular cytotoxicity and Treg ratios in mouse spleen

**Figure 4:**
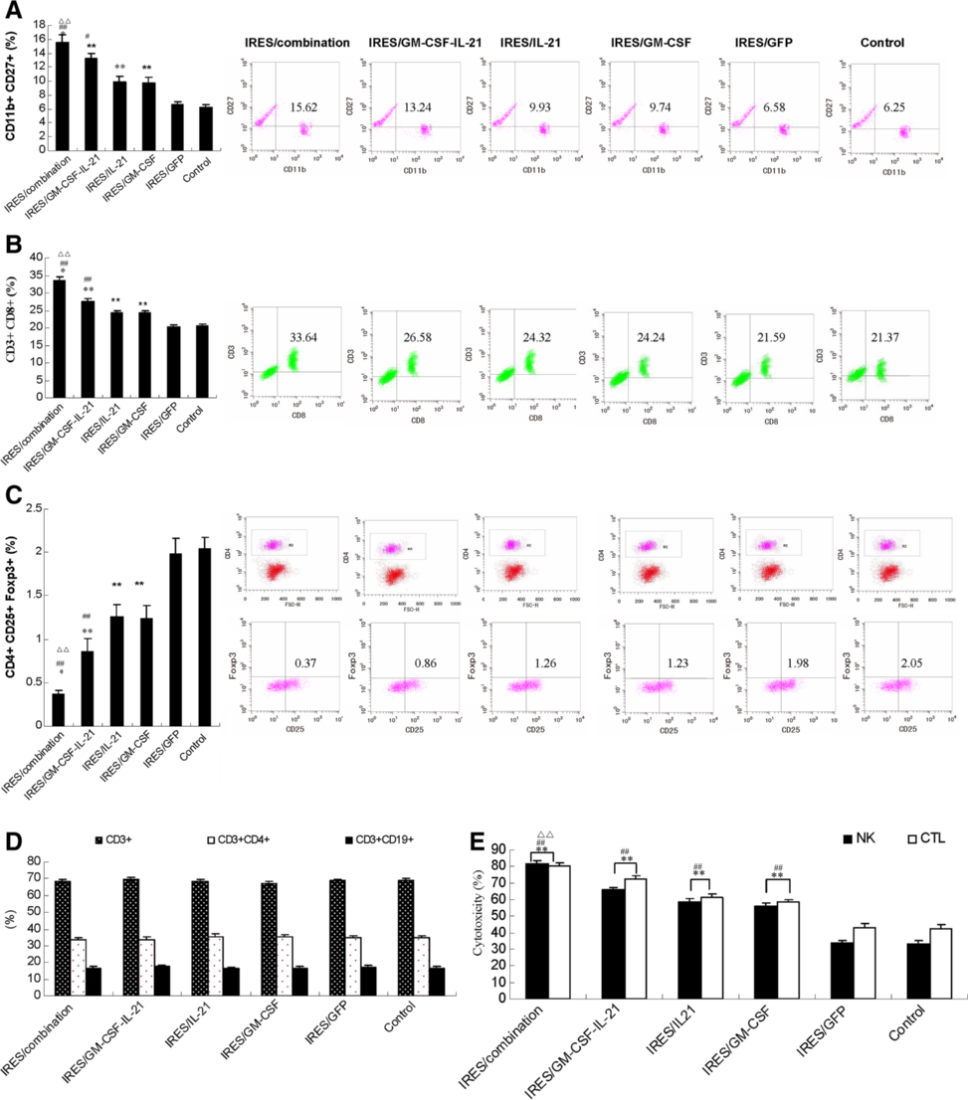
**Effect of the plasmids on NK, CTL and regulatory T cell (Treg) frequencies in mice. ** Plasmids included: control, pGFP-IRES, pGM-CSF-IRES, pIL-21-IRES, pGM-CSF-IRES- IL-21, and pGM-CSF-GFP-IRES-Rae-1-IL-21. At 10 d after treatment, mice were sacrificed and flow cytometry was used to determine splenic total T cells CD3+, Th cells (CD3+ CD4+), CTL (CD3+ CD8+), B cells (CD3-CD19+), NK cells (CD11b + CD27+) and the proportion of Treg cells (CD4+ CD25+ Foxp3+). **A**: flow chart and frequency of NK, and **B**: flow chart and frequency of CTL in a mixed population of spleen cells. **C**: flow chart and frequency of Treg cells from the same tissue. **D**: ratios of T cells (CD3+), Th cells (CD3+ CD4+), and B cells (CD3-CD19+) in the spleen after 10 d of treatment. **E**: NK and CTL-mediated cytotoxity as determined by MTT assays after 10 d of treatment. All data were expressed as mean ± standard deviation (n = 3). Note: ^*^P < 0.05 and ^**^P < 0.01, as compared with control. ^#^P < 0.05 and ^##^P < 0.01, as compared with IRES/GM-SCF and IRES/IL-21. ^△^P < 0.05 and ^△△^P < 0.01, as compared with IRES/GM-SCF-IL-21.

**Figure 5:**
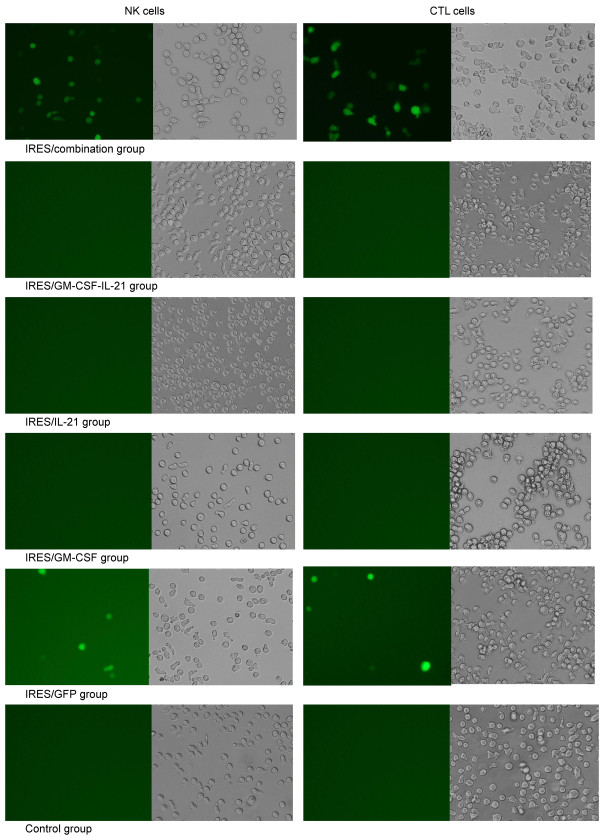
**CTL and NK cells were observed by fluorescence microscopy (n = 3).** 10 d after treatment, mice spleens were harvested, NK and CTL cells were elected by flow cytometry. Hepatic cancer cells carried the green fluorescent protein (GFP)-expressing vector, and upon injury by CTL and NK cells, the carrying GFP gene vectors is released from the cells in hepatic cancer tissues. GFP-expressing NK and CTL cells were observed by scanning with a fluorescence microscope (× 200 magnification). The right panel represents the phase contrast images of transfected CTL and NK cells that expressed the GFP-fluorescent marker.

In order to study the putative mechanisms responsible for the inhibitory effects of the pGM-CSF-GFP-IRES-Rae-1-IL-21 construct on liver cancer tumors, we measured spleen cell frequencies by flow cytometry and MTT assays. We determined that the proportion of CD3-CD19+, CD3+ CD4+ and CD3+ cells in mouse spleen were not significantly (P > 0.05) different before or after treatment (Figure 4D). The proportion and cytotoxicities of both NK and CTL cells were significantly (P < 0.01) different among the six groups (see Figure 4A-B, and Table 2). When compared with the control group, the frequency and cytotoxicity of NK and CTL cells were significantly (P < 0.01) increased in the IRES/combination, IRES/GM-SCF-IL-21, IRES/IL-21, and IRES/GM-SCF groups, especially in respect of the IRES/combination. The proportion and cytotoxicity of NK and CTL cells were significantly (P < 0.01) higher in the IRES/GM-SCF or IRES/GM-SCF-IL-21 groups than in the IRES/IL-21 group. There was no significant (P > 0.05) difference between either the control or the IRES/GFP group, or between the IRES/IL-21 and IRES/GM-SCF groups. The CTL and NK cells showed green fluorescence in the IRES/combination and IRES/GFP group (Figure 5), and the fluorescence intensity of both CTL and NK cells in the IRES/combination group was significantly stronger as compared with the IRES/GFP group. In addition, there was no green fluorescence in other groups because they lacked the GFP gene. These observations further confirmed the effect of NK and CTL cells against targeted tumor cells.

**Table 2:** The raw data showing the frequency of NK, CTL and Treg cells in each group (n = 3)

**Group**	**CD11b + CD27 + (%)**		**CD3 + CD8 + (%)**		**CD4 + CD25 + Foxp3 + (%)**	
	**NK**		**CTL**		**Treg cells**	
IRES/combination group	16.24	15.60 ± 0.66	34.58	33.60 ± 1.00	0.41	0.37 ± 0.04
	15.62		33.64		0.37	
	14.95		32.59		0.33	
IRES/GM-CSF-IL21 group	13.78	13.40 ± 0.33	27.93	26.59 ± 1.33	1.01	0.87 ± 0.14
	13.24		26.58		0.86	
	13.18		25.27		0.73	
IRES/IL21 group	10.32	10.03 ± 0.26	24.78	24.36 ± 0.41	1.34	1.26 ± 0.07
	9.93		24.32		1.26	
	9.83		23.97		1.19	
IRES/GM-CSF group	9.97	9.80 ± 0.15	24.79	24.07 ± 0.82	1.32	1.21 ± 0.12
	9.74		24.24		1.23	
	9.69		23.18		1.09	
IRES/GFP group	6.72	6.60 ± 0.11	21.97	21.61 ± 0.36	2.01	1.95 ± 0.08
	6.58		21.59		1.98	
	6.51		21.26		1.86	
Control group	6.45	6.36 ± 0.10	21.73	21.46 ± 0.24	2.20	2.08 ± 0.11
	6.25		21.37		2.05	
	6.37		21.28		1.98	

At the end of treatment, the frequencies of Tregs were significantly different among groups (Figure 4C and Table 2). When compared with control, Tregs were significantly (P < 0.01) fewer in number in the IRES/combination groups, IRES/ GM-SCF-IL-21, IRES/IL-21 and IRES/GM-SCF groups. Tregs were also significantly (P < 0.01) lower in the IRES/GM-SCF- IL-21 group than in either the IRES/IL-21 or the IRES/GM-SCF groups. No significant (P > 0.05) difference was found between either the IRES/IL-21 group or the IRES/GM-SCF group, or between the control and IRES/GFP groups. These observations indicated that pGM-CSF-GFP-IRES-Rae-1-IL-21 significantly inhibited the growth of liver tumors by lowering the Treg frequency, increasing immune modulation, frequency and cytotoxic activities of immune cells like CTL and NK cells, enhancing Rae-1 expression in liver cancer tissues, and facilitating the recognition of tumor cells by CTL and NK cells and debulking liver cancer.

## Discussion

The development of tumors is related to the relative competence of host immunity [16]. As with other tumors, liver cancer induces a relative state of immune suppression or dampening of host immunity, and a change in the molecular expression of surface markers by cancer cells that permits the escape of tumor cells from immune recognition [17] by such cells as NK and CTL cells. Meanwhile, in tumor cells that are recognized by CTL and NK cells (e.g., MHC-1 ligand and Rae-1) obviously decrease or disappear, and liver cancer cells survive in the host [18,19]. Current studies on the treatment of tumors by GM-SCF or IL-21 mostly focus on enhancing CTL and NK cell-mediated activity [20,21] or increasing genes that can be expressed by tumors and recognized by NK and CTL cells in tumor tissues [22]. Such strategies rarely endeavor to simultaneously increase cell-mediated activity or genes that are recognized by NK and CTL cells in tumors.

In this study, we successfully constructed an immune escape inhibition system (Figure 1A), which was afforded by the construction of the recombinant expression plasmid pGM-CSF-GFP-IRES- Rae-1-IL-21 that passed RT-PCR and gene sequencing analyses to augment host immunity and proteins that are expressed by tumor cells and then recognized by host immune cells. The recombinant plasmid (IRES/combination) used in this mouse model of subcutaneous liver cancer resulted in decreased tumor weights as compared mice that were transfected with IRES/GM-SCF-IL-21, IRES/GM-SCF and IRES/IL-21 treatment alone. In the IRES/combination transfected animals, the tumor volume was the least by the end of treatment. By 1–5 d after treatment, the tumor volume in each group gradually increased, which might be related to a weak inhibition of the tumor caused by low gene expression at the beginning of treatment. By 6–10 d after treatment, the tumor volume in the IRES/combination group was decreased, which might be associated with a potent anti-tumor effect caused by high expression of the target genes. In the other groups, the tumor volume increased at a much lower rate or even decreased. This observation indicated an inhibitory effect of these genes on tumor size. Among the groups, the anti-tumor inhibitory effect of the IRES/combination was highest and tumor cell necrosis, monocyte- and neotrophilic-infiltration were more obvious in the tumor tissues (Figure 2A(f)). We also found that the survival rates of mice were highest in the IRES/combination group, which indicated that recombinant genes of GM-SCF, IL-21, and Rae-1 displayed the most effective treatment against liver cancer.

Cytokines belong to small active molecules produced by immune cells. Cytokines have potent immune-regulating effects, and play important roles in tumor immunity [23,24]. GM-SCF and IL-21 augment expression of INF-γ and IL-2, improve the activity of CTL and NK cells, and inhibit growth of tumors [25,26]. Our study showed that levels of both INF-γ and IL-2 in mice treated with recombinant GM-CSF, IL-21 and Rae-1 gene expression vectors were highly expressed and negatively associated with the tumor volumes seen in tumor-bearing mice. The numbers and activity of CTL an NK cells in the IRES/combination groups were higher than was seen in the IRES/GM-SCF-IL-21, IRES/GM-SCF, and IRES/IL-21 groups, indicating that the GM-SCF, IL-21 and Rae-1 genes had a synergistic effect in promoting secretion of INF-γ and IL-2 levels, and the numbers and activity of CTL and NK cells. INF-γ is secreted by CTL and NK cells and belongs to the glycoprotein family. INF-γ displays potent anti-viral effects, and is involved in immune regulation, has anti-proliferative effects, displays other biological characteristics [27,28] and directly inhibits tumor cells. INF-γ displays immuno-regulatory effects and activates enhanced phagocytic functions of both mononuclear macrophages and NK cells. INF-γ also enhances the differentiation and maturation of B and T cells, increases the cytotoxic activity of CTL cells, induces the expression of MHC-I antigens by tumor cells, and increases the recognition of tumors by immune cells such as CTL and NK cells [29].

The cytokine IL-2 is a growth factor of T cells, and is important in the activation and proliferation of T cells, activates B cells and macrophages, and activates T cells to secrete IL-2 [30]. Therefore, in this study, the combination of GM-SCF, IL-21 and Rae-1 collectively activated the murine immune system in liver cancer, and increased the numbers and cytotoxic activities of NK and CTL cells. Activated CTL and NK cells secreted INF-γ, and high INF-γ levels enhanced the numbers and cytotoxic activities of NK and CTL cells. Likewise, the release of IL-2 promoted significant activation of CTL in an autocatalytic immunological cycle that finally inhibited tumor growth.

We found that the expression of Rae-1 and protein was significantly higher in the IRES/combination group as compared with the other groups, and that GM-SCF and IL-21 stimulated the expression of the Rae-1 gene. Most NK and CTL cells express the Rae-1 receptor, which easily combines with Rae-1 that is expressed by liver cancer tissues to enhance the removal of cancer tissue by NK cells and CTL [31]. Our study showed that pGM-CSF-IL-21-Rae-1 increased the number and cytotoxicity of CTL and NK cells, and enhanced the expression of Rae-1 in liver cancer tissues. Therefore, CTL and NK cells that expressed the Rae-1 receptor efficiently eliminated Rae-1.

Tregs belong to a subgroup of T cells that are of special immune regulatory importance, and are important in tumor immunity. Foxp3 is a special transcription factor expressed by Tregs and it is essential for their development and function [32]. First, as with other tumors, liver cancer causes an increase in the frequency of Tregs and immune inhibition by non-specific suppression of major histocompatibility complex (MHC), and second it causes specific suppression. In the first mechanism, there is broad inhibition of immunity including effects on dentritic cells, NK cells, macrophages, and CD4+ and CD8+ T cell sub-populations [33,34]. In specific suppression, Treg is of special T cell receptor immune complex (TCR) importance and displays specific MHC restriction. Thus, antigens related to specific tumors activate Tregs. Active Tregs inhibit activation of CD4+, CD8+ T cells and B cells, which can finally result in the resistance of immune cells to tumor antigens [35]. In this study, we treated liver cancer with a recombinant plasmid of GM-SCF, IL-21 and Rae-1 and found that the frequency of Tregs was significantly decreased as compared with treatment using a single gene. This observation indicated that the recombinant plasmid played a synergistic effect on the decrease in Treg frequencies. It was previously reported that IL-21 enhances activation and cytotoxicity of CTL and NK cells, and alleviates immune inhibition of Tregs [36,37]. As an anti-apoptotic cytokine, IL-21 can sustain survival of active T cells [36,37]. Tian Yongju [38] found that IL-21 decreases Treg frequencies and apoptosis of peripheral blood mononuclear cells (PBMC), and greatly increases the cytotoxicity of PBMC, which can be alleviated by IL-21 antibodies. However, thus far, little information has detailed the effects of GM-SCF and Rae-1 gene expression on Treg frequencies in a mouse tumor model. Here, we have attempted to confirm that IL-21 decreased the frequency of Tregs. Further, this effect was promoted by expression of GM-SCF and Rae-1. The mechanisms involved in this effect require further study.

## Conclusion

In summary, GM-SCF, IL-21 and Rae-1 greatly inhibited the growth of liver cancer in a relevant mouse model. The mechanisms responsible for this inhibitory effect might be related to the activation of host cell-mediated immunity that was induced by the triple gene expression vector. We also propose that this might have increased the numbers and cytotoxicity of NK and CTL cells, lowered Treg frequency, and alleviated any inhibition of CTL and NK cells. Active CTL and NK cells increased their ability to secrete INF-γ that augments activation and cytotoxicity of NK and CTL cells. Similarly, active CTLs increased their ability to secrete IL-2 that can drive activation and cytotoxicity of CTL. In an autocatalytic cascade of cell-mediated immunity and immune pathways, the recombinant triple-gene expression plasmid increased Rae-1 expression in cancer, thus efficiently eliminating this tumor by the combined activities of CTL and NK cells that express cell membrane-associated Rae-1 receptors.

## Competing interests

The authors declare that they have no competing interests.

## Authors’ contributions

MC and BH participated in the study design, performing experiments, data analysis and drafting of the manuscript. KZ and XG performed *in vitro* experiments and data analysis. YL and JH participated in the study design and manuscript editing. ZZ and YW were involved in the conception and design of the study, data preparation and analysis, manuscript drafting and revisions. All authors read and approved the final draft of the manuscript.
